# Comparative Analysis of Gut Microbiome Composition and Blood Lipid Profiles in Intensively Reared Broiler Chickens and Ducks

**DOI:** 10.3390/ani16081240

**Published:** 2026-04-17

**Authors:** Zsombor Szőke, Njomza Gashi, Péter Dávid, Péter Fauszt, Maja Mikolás, Emese Szilágyi-Tolnai, Endre Szilágyi, Piroska Bíróné Molnár, Georgina Pesti-Asbóth, Judit Rita Homoki, Ildikó Kovács-Forgács, Ferenc Gál, László Stündl, Judit Remenyik, Melinda Paholcsek

**Affiliations:** 1 Center for Complex Systems and Microbiome Innovations, Faculty of Agricultural and Food Sciences and Environmental Management, University of Debrecen, H-4032 Debrecen, Hungary; njomza.gashi@agr.unideb.hu (N.G.); david.peter@agr.unideb.hu (P.D.); fauszt.peter@agr.unideb.hu (P.F.); mikolas.maja@agr.unideb.hu (M.M.); tolnai.emese@agr.unideb.hu (E.S.-T.); szilagyi.endre@agr.unideb.hu (E.S.); molnar.piroska@agr.unideb.hu (P.B.M.); georgina.asboth@agr.unideb.hu (G.P.-A.); homoki.judit@agr.unideb.hu (J.R.H.); forgacs.ildiko@agr.unideb.hu (I.K.-F.); drgalferencgabor@gmail.com (F.G.); remenyik@agr.unideb.hu (J.R.); 2 Department of Food Technology with Biotechnology, Faculty of Agriculture and Veterinary, University of Prishtina, 10 000 Prishtina, Kosovo; 3 Institute of Food Technology, Faculty of Agricultural and Food Sciences and Environmental Management, University of Debrecen, H-4032 Debrecen, Hungary; stundl@agr.unideb.hu

**Keywords:** poultry, intensive production, gut microbiome, microbiome maturation, functional pathways, lipid metabolism, host–microbiome interactions

## Abstract

This study investigated phase-dependent changes in gut microbiome composition, predicted functional potential, and lipid metabolism in intensively reared broiler chickens and ducks across the starter, grower, and finisher phases (from day-old to 42 days of age), over six production cycles (four chicken and two duck cycles), using 16S rRNA sequencing and blood lipids profiling. A total of 70 pooled manure samples were collected (46 from chickens and 24 from ducks), along with 34 blood samples (22 from chickens and 12 from ducks), all obtained under standard production conditions. Microbial diversity remained stable across growth phases within each species, whereas clear interspecies differences were observed (*p* < 0.01). Microbiome maturation involved a shift from early facultative and environmentally associated taxa during the starter phase (day-old to 14 days of age), including *Acinetobacter* (*p* < 0.01) and *Enterococcus* (*p* < 0.001), toward a more stable, host-adapted community. At the level of predicted functional pathways, shifts between growth phases were more pronounced in ducks. Predicted gene-level profiles showed phase-specific differentiation in chickens, with starter-associated genes linked to core carbon and nitrogen metabolism and finisher-associated genes related to structural and transport functions, whereas ducks exhibited a more balanced reorganization involving carbohydrate, energy, and nitrogen metabolism. Host lipid profiles between adjacent growth phases showed dynamic shifts in ducks (*p* < 0.05). These species-specific lipid patterns were mirrored by microbiome–lipid associations, as demonstrated by correlation analyses between dominant bacterial genera and blood lipid parameters, revealing more coordinated relationships in chickens and more heterogeneous patterns in ducks. Overall, these findings demonstrate species-specific organization of gut microbiome changes and their integration with blood lipid profiles under intensive production conditions.

## Simple Summary

Understanding how gut microorganisms change during growth is important for improving poultry production. In this study, we examined two of the most important poultry species, broiler chickens and ducks, raised under intensive farming conditions. Our aim was to compare how their gut microbial communities develop over time and how these changes relate to blood lipid levels, which are linked to metabolism. We found that although overall microbial diversity remained relatively stable, clear differences existed between species. As the animals grew, their gut communities shifted from early, environment-associated bacteria toward more stable and specialized microorganisms. These changes were also linked to differences in blood lipid profiles, with ducks showing more dynamic patterns and chickens more coordinated relationships. The results provide practical insight into how microbiome changes are associated with metabolism in poultry, based on predicted functional activity. These findings may support the development of feeding and management strategies aimed at improving animal health and production efficiency.

## 1. Introduction

Poultry meat is one of the most important and widely consumed animal protein sources worldwide, largely due to its affordability and broad cultural acceptance. Driven by global population growth and urbanization, the demand for poultry meat continues to increase, positioning the sector as a crucial contributor to future protein supply [1]. Global poultry meat production has increased dramatically from about 9 million tonnes in 1961 to over 133 million tonnes in 2020, now representing nearly 40% of all meat produced worldwide [2]. This rapid growth, driven by rising population and urbanization, highlights the sector’s intensification and its critical role in meeting future protein demand.

As demand continues to rise, poultry production has become increasingly intensive, with a strong focus on delivering safe, efficient, and affordable poultry meat [3]. With the increasing intensification of poultry production, maintaining efficiency and product quality requires careful attention to biological factors that influence overall performance. One of the most influential among these is the poultry gut microbiome, which plays a central role in digestion, metabolism, immune function, and general health [4,5,6].

As birds progress through their rearing phases, the composition of the gut microbiome undergoes marked age-dependent shifts before reaching a more established community. Due to the characteristically short gastrointestinal tract and rapid digesta transit in poultry, their microbial profile differs distinctly from that of mammals, leading to pronounced changes in microbial composition across production stages [4]. These shifts are not only structural but also functional, as age and diet-dependent alterations in the microbiota also reshape the metabolic outputs they generate [7]. Through its metabolic activity, the gut microbiota modulates several metabolic processes, including the host’s energy homeostasis, glucose metabolism [8], and lipid metabolism [9]. Given that these metabolic processes ultimately shape systemic physiology, their effects are also reflected in circulating blood biochemical parameters. These parameters provide an objective snapshot of an animal’s physiological state, revealing both metabolic and nutritional conditions. Key indicators such as cholesterol (CHOL), triglycerides (TGs) and their lipoprotein fractions are closely linked to growth performance, as they reflect essential aspects of lipid metabolism. In circulation, cholesterol is transported mainly as high-density (HDL-CH) and low-density lipoproteins (LDL-CH), which together provide a clear picture of systemic lipid status. Cholesterol homeostasis is critical for normal cellular and systemic functions, and disturbances in cholesterol levels can impair animal health and productivity. Elevated cholesterol levels in poultry have been associated with reduced reproductive performance and it also increases susceptibility to metabolic disorders such as fatty liver and arteriosclerotic changes [10]. Poultry lipid metabolism is closely tied to triglyceride levels, which play a central role in abdominal fat deposition and overall energy storage. Persistently elevated triglycerides are linked to metabolic disturbances, such as fatty liver development and reduced insulin sensitivity that can compromise both the health and productive performance of poultry [11].

Although research on poultry gut microbiota has expanded considerably, significant gaps remain in understanding how microbiome development, functional potential, and host metabolic responses interact across different growth phases, particularly under intensive production systems. Existing studies have largely examined microbial composition or host physiology in isolation, while approaches that connect microbiome structure, predicted functional pathways, and systemic metabolic indicators are still limited. Furthermore, comparative studies between poultry species raised under standardized conditions are relatively scarce. Bridging these gaps is crucial for advancing our understanding of microbiome-mediated metabolic regulation, with important implications for animal health, production efficiency, and sustainable poultry systems. To address these gaps, this study pursued the following objectives (i) to characterize changes in blood lipid parameters (HDL, LDL, total CHOL, and TRIGL) across the starter, grower, and finisher phases in chickens and ducks; (ii) to analyze gut microbiome composition using 16S rRNA metagenomic sequencing, mapping diversity indices throughout development in both species and comparing their microbiome structures; (iii) to assess correlation patterns between the top 30 most abundant bacterial genera and lipid parameters in chickens and ducks in order to identify microbiota-lipid metabolism interactions; (iv) to predict functional pathways and gene-level profiles based on 16S rRNA data, providing preliminary insights into microbiome-associated metabolic potential.

## 2. Materials and Methods

### 2.1. Birds and Housing

**Figure 1:**
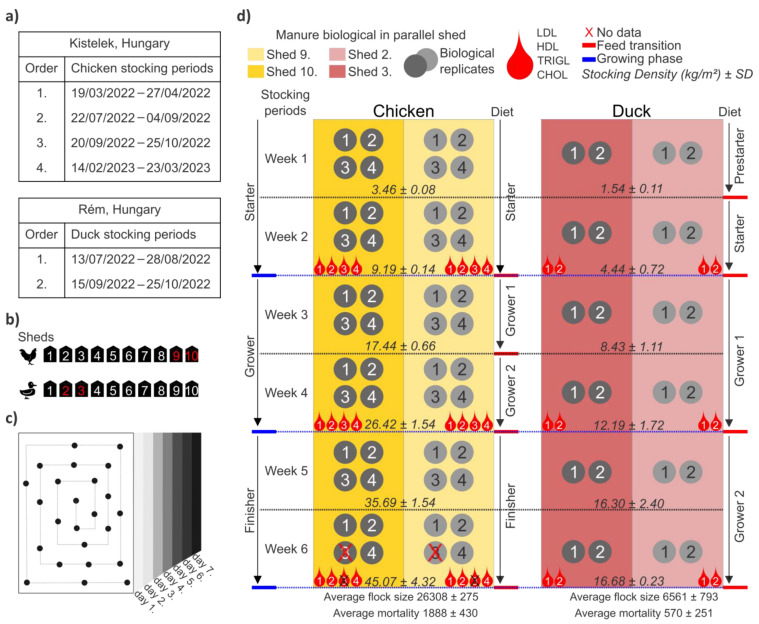
Overview of the study. (**a**) Stocking period of broiler chickens and ducks during a six-week production cycle. (**b**) Experimental sheds included in this study (2–3 and 9–10), representing biological replicates for each species. The sheds highlighted in red indicate those included in the analysis. (**c**) Representative manure sampling using a circular walkthrough pattern with daily collection and weekly pooling. (**d**) Experimental timeline showing production phases (starter, grower, finisher), feed transition for ducks (Duck-starter, Duck-grower I, Duck-grower II) and for chickens (Chick-starter, Chick-grower I, Chick-grower II, Chick-finisher), manure and blood sampling time points. Colors distinguish species and biological replicates. Average flock size, stocking densities and mortality are indicated. The nutritional composition of the feed is provided in Supplementary File S1.

The samples were collected from the sites of Hungerit Zrt. (GPS coordinates: 46.65513817352346, 20.271366227943012). Intensively reared poultry species (Ross 308 broilers, Cherry Valley ducks) were also examined between 2022 and 2023. Over the one-year study spanning six production cycles, four broiler chicken cycles and two duck cycles were monitored daily throughout their production phases. Both broilers (shed 9–10) and ducks (shed 2–3) were kept in two parallel sheds and reared under standard management conditions in thermostatically controlled houses. The farm operates its own breeding stock and hatchery. As a result, day-old poultry (both ducks and chickens) are produced in-house following standardized protocols and are delivered to rearing farms under strictly controlled conditions. Ducks underwent a feed transition within the starter phase, moving from ‘Duck-pre-starter’ to ‘Duck-starter’ feed after one week, followed by ‘Duck-grower I’ and ‘Duck-grower II’ feeds during the grower and finisher stages, respectively. Broilers, in contrast, were fed ‘Chick-starter’ feed throughout the starter phase, transitioning between ‘Chick-grower I’ and ‘Chick-grower II’ feeds during the grower stage, and receiving ‘Chick-finisher’ feed exclusively in the final phase. The total average number of broilers was 26,308 per stocking period. Chickens were kept in floor pens covered with wood shavings. Average stocking densities varied during the different rearing phases (stage 1 (3.46 kg/m^2^), stage 2 (9.19 kg/m^2^), stage 3 (17.44 kg/m^2^), stage 4 (26.42 kg/m^2^), stage 5 (35.69 kg/m^2^), stage 6 (45.07 kg/m^2^)). The total average number of ducks was 6561 per stocking period. Ducks were kept in floor pens covered with wood shavings. Average stocking densities varied during the different rearing phases (stage 1 (1.54 kg/m^2^), stage 2 (4.44 kg/m^2^), stage 3 (8.43 kg/m^2^), stage 4 (12.19 kg/m^2^), stage 5 (16.30 kg/m^2^), stage 6 (16.68 kg/m^2^)) (Figure 1).

### 2.2. Sample Collection

Continuous monitoring was carried out at four stocking periods per broiler (chicken 1st, 2nd, 3rd, 4th) and two stocking periods per duck (duck 1st, 2nd). To enable accurate comparison between production systems (across intensively reared poultry), both were standardized into three distinct phases, starter (day 0–14), grower (day 15–28), and finisher (day 29–42), each lasting two weeks. Due to species-specific and overlapping feeding schemes across production phases (Figure 1), the effect of feed was not evaluated separately, and samples were pooled and analyzed based on age, allowing the assessment of age-related changes in microbiome and blood lipid parameters.

#### 2.2.1. Biological-Footbag (Manure) Samples

Due to the hygienic criteria of the livestock farm and poultry barn, and the maintenance of a sterile environment (pathogens outside the farm), fecal samples were collected with textile footbags that fit the footwear. During daily sampling, poultry barns were carefully walked around to ensure homogeneous sampling. Biological-footbag (manure) samples were stored in individually marked lockable sterile bags and transported on ice to the laboratory of the University of Debrecen. The samples were stored at −80 °C until pooling and DNA extraction. Biological-footbag (manure) samples were collected at 1, 2, 3, 4, 5 and 6 weeks of age. The biological-footbag samples were pooled by week (±1 day), with each pool consisting of average of 7 subsamples (one per day). During the experiment 70 manure samples were collected, a total of 46 pooled manure samples from chicken barns. A total of 24 pooled manure samples from duck barns. Sampling in broilers was unsuccessful during the final week of the third production cycle in both sheds (9 and 10), resulting in missing samples for this time point (Figure 1).

#### 2.2.2. Blood Samples

Blood samples were collected biweekly at the end of each production phase (starter day 14, grower day 28, and finisher day 42), corresponding to weeks 2, 4, and 6 (±1 day). To ensure representativeness, each measurement was conducted in 10 replicates, based on a total of 34 blood sampling events, 22 conducted in broilers and 12 in ducks (biweekly sampling, with one blood sample measured in 10 replicates and averaged). Similarly, blood sampling in broilers was unsuccessful during the final week of the third production cycle in both sheds (9 and 10), resulting in missing samples for this time point. The samples were collected in vacutainer tubes with K3EDTA (potassium-ethylenediaminetetraacetic acid) anticoagulant, with each replicate individually labeled for traceability. Following collection, blood sample was collected and transported to the laboratory on ice and stored at −80 °C until measurement. A total of four blood parameters were analyzed (low-density lipoprotein (LDL), high-density lipoprotein (HDL), triglycerides (TRIGL), and cholesterol (CHOL)) (Figure 1).

### 2.3. Microbiome Methods

#### 2.3.1. Sample Preparation and Mechanical Cell Lysis

Bacterial cell suspensions were made from biological-footbag samples (manure samples). Each pooled sample consisted of seven subsamples. In the first step, the samples were washed in sterile PBS (Biosera, Cholet, France) to obtain a uniform bacterial suspension. Subsequent steps were carried out according to the manufacturer’s instructions with minor modifications, as previously described [12].

#### 2.3.2. DNA Extraction

DNA extraction from bacterial cell suspensions was performed using the QIAamp Fast DNA Stool Mini Kit (Qiagen, Hilden, Germany, Cat. No. 51604), and subsequent DNA quality assessment was carried out according to the manufacturer’s instructions with minor modifications, as previously described [12].

#### 2.3.3. Library Preparation, Sequencing

DNA library preparation and sequencing were performed in accordance with the Illumina 16S Metagenomic Sequencing Library Preparation protocol (15044223 Rev. B) and followed the workflow outlined by Skopkó [13]. Each reaction was initiated with 12.5 ng of stool-derived DNA. The V3-V4 regions of the bacterial 16S rRNA gene were amplified using the universal primers 341F (5′-CCTACGGGNGGCWGCAG-3′) and 785R (5′-GACTACHVGGGTATCTAATCC-3′), both equipped with Illumina overhang adapters. Amplicons of approximately ~460 bp were produced using 2× KAPA HiFi HotStart ReadyMix, then indexed with the Nextera XT Index Kit and purified with MagSI Pure Beads, resulting in libraries ranging from roughly 550 to 630 bp. Library quality was verified using an Agilent D1000 ScreenTape system, quantified by qPCR, then normalized and combined in equimolar amounts. The pooled library (4 nM) was denatured with 0.2 M NaOH, diluted to a final concentration of 8 pM, and sequenced on an Illumina MiSeq platform with the MiSeq Reagent Kit v3 (600 cycles) following standard protocols.

### 2.4. Blood Measurement

Blood samples were collected into vacutainer tubes containing K_3_EDTA (potassium ethylenediaminetetraacetic acid) as an anticoagulant. Following blood collection, the samples were centrifuged at 2500 rpm for 10 min at room temperature. The resulting plasma was then stored at −80 °C until further analysis. Before each measurement, calibration (C.f.a.s. Lipids (System ID 07 6570 8); C.f.a.s. (System-Code 401)) and quality control (PreciControl ClinChem Multi 1; 2 (System ID 07 7469 3; 07 7470 7)) were performed according to manufacturer guidelines (Roche cobas c311 analyzer, Hitachi High-Technologies Co., 24-14, Tokyo, Japan).

#### 2.4.1. LDL Enzymatic Colorimetric Assay

LDL-associated cholesterol esters and free cholesterol were quantified using a cholesterol enzymatic assay based on cholesterol esterase and cholesterol oxidase. Surfactants selectively solubilized LDL particles, while enzymatic reactions with other lipoproteins (HDL, VLDL, chylomicrons) were inhibited by specific surfactants and a sugar compound. The resulting colorimetric signal, measured at 583/659 nm, was directly proportional to LDL concentration. Sample volume was 2 μL (test LDLC3, test ID 0-452).

#### 2.4.2. HDL Enzymatic Colorimetric Assay

HDL cholesterol was measured using an enzymatic colorimetric assay. Non-HDLs (LDL, VLDL, chylomicrons) were inactivated by forming water-soluble complexes with polyanions and detergents, preventing their reaction with cholesterol esterase and cholesterol oxidase. Only HDL particles participated in the enzymatic reactions. Cholesterol esters were hydrolyzed to free cholesterol and fatty acids, and the colorimetric signal measured at 583/800 nm was directly proportional to HDL concentration. Sample volume was 2.5 μL (test HDLC4, test ID 0-389).

#### 2.4.3. Triglyceride Enzymatic Colorimetric Assay

Triglyceride levels were determined enzymatically based on the method of Wahlefeld, employing microbial lipoprotein lipase for complete hydrolysis of triglycerides to glycerol. Glycerol was then oxidized to dihydroxyacetone phosphate and hydrogen peroxide, which reacted with 4-aminophenazone and 4-chlorophenol in the presence of peroxidase, forming a red dye (Trinder reaction). The color intensity, measured at 700/505 nm, was directly proportional to triglyceride concentration. Sample volume was 2 μL (TRIGL 250 tests System-ID 07 6710 7).

#### 2.4.4. Cholesterol Enzymatic Colorimetric Assay

Total cholesterol was measured enzymatically. Cholesterol esters were hydrolyzed by cholesterol esterase to free cholesterol and fatty acids. Cholesterol oxidase then converted cholesterol to cholest-4-en-3-one and hydrogen peroxide. In the presence of peroxidase, hydrogen peroxide reacted with phenol and 4-aminoantipyrine (4-AAP) to form a red quinone imine dye. The color intensity, proportional to cholesterol concentration, was measured at 700/505 nm. Sample volume was 2 μL (CHOL Gen.2 System-ID 07 6726 3).

### 2.5. Statistical Analysis and Data Visualization

Raw 16S rRNA gene sequencing data were processed using a QIIME2-based bioinformatics pipeline, as previously described [13], and the resulting feature table was imported into R for downstream analyses. Prior to downstream analyses, data were filtered, normalized, and transformed using the phyloseq package. Continuous variables are presented as mean ± standard deviation. All statistical analyses and data visualizations were performed in RStudio (v2025.09.0 + 387, Posit Software, 2025, Boston, MA, USA). Data distribution was assessed using the Shapiro–Wilk test before statistical analysis. Given the limited sample size and the presence of variables showing deviations from normality, differences between growing phases were evaluated using the non-parametric Wilcoxon rank-sum test (starter vs. grower, grower vs. finisher, starter vs. finisher) for all measured variables (including blood parameters and microbiome-derived variables). Statistical significance was defined as *p* < 0.05. Alpha diversity metrics (Shannon) were computed using the phyloseq v.1.44 package in R software [14,15]. Functional profiles were predicted from ASVs using PICRUSt2, which includes phylogenetic placement of ASVs, hidden-state prediction of gene family abundances, and inference of metabolic pathways [16]. All outputs represent predicted functional potential rather than directly measured activities. Predicted gene families and metabolic pathways were annotated for functional classification by mapping to Kyoto Encyclopedia of Genes and Genomes (KEGG) identifiers. No copyrighted KEGG pathway maps or related materials were included or reproduced [17]. Differential abundance analyses of microbial taxa and predicted functional pathways were performed using DESeq2, and the resulting contrasts were visualized as volcano plots generated with ggplot2. Correlations between microbial taxa (genera) and blood biochemical parameters were assessed using Spearman’s rank correlation with the corrplot R package (version 0.92) [18] and visualized as heatmaps created with the pheatmap package (version 1.0.12) [19]. LEfSe (Linear Discriminant Analysis Effect Size) analysis was performed in R using the phyloseq (v1.46.0) and microbiomeMaker (v1.13.2) packages. An LDA score ≥ 4 was used as the threshold for biological relevance, with statistical significance determined by Wilcoxon test (*p* < 0.05). Venn diagrams were constructed using the limma R package (version 3.28.14) [20]. The graphs were produced in ggplot2 and related R packages (version 3.5.0) [21,22].

For microbiome analyses, growing phases (starter, grower, and finisher) were compared. Due to weekly sampling from parallel sheds across stocking periods, the broiler starter and grower phases each consisted of 16 samples (*n* = 16), while the finisher phase included 14 samples (*n* = 14) due to missing samples. Under the same sampling design, the duck starter, grower, and finisher phases each consisted of 8 samples per phase (*n* = 8) (Figure 1). For blood analyses, growing phases (starter, grower, and finisher) were also compared. Due to sampling performed every second week from separate sheds across stocking periods, the broiler starter and grower phases each consisted of 8 samples (*n* = 8), while the finisher phase included 6 samples (*n* = 6) due to missing samples. Under the same sampling design, the duck starter, grower, and finisher phases each consisted of 4 samples per phase (*n* = 4) (Figure 1). The raw blood parameters data are provided in Supplementary File S2.

## 3. Results

**Figure 2:**
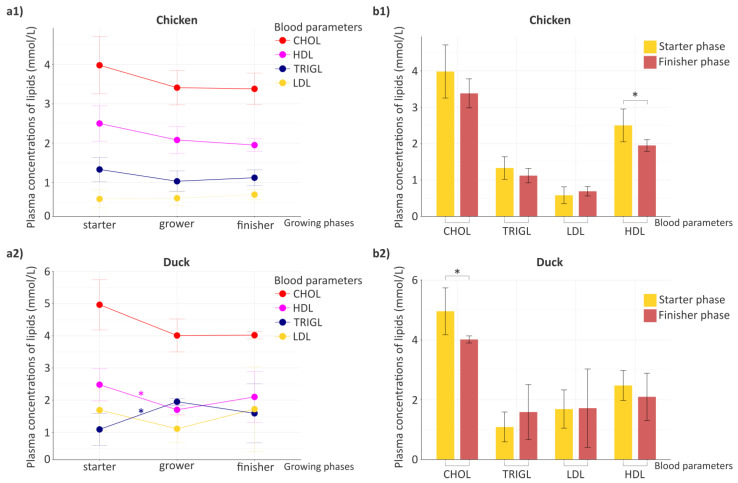
Developmental changes in key blood lipid parameters across growing phases. (**a**) Line plots illustrate phase-specific trajectories of major blood lipid parameters (high-density lipoprotein (HDL), low-density lipoprotein (LDL), total cholesterol (CHOL), triglycerides (TRIGL)) across growing phases (starter, grower, finisher) highlighting their developmental trends over time, separately for chicken (**a1**) and duck (**a2**). (**b**) Bar charts present simplified comparisons of the same lipid parameters between the starter and finisher phases, enabling a direct assessment of early and late stage differences, separately for chicken (**b1**) and duck (**b2**). Color coding distinguishes growing phases and lipid parameters. For line plots, colors indicate the measured lipid parameters (red = CHOL, purple = TRIGL, blue = LDL, yellow = HDL). For bar charts, yellow represents the starter phase and red represents the finisher phase. Asterisks indicate significant differences (* *p* < 0.05). The summary blood parameter values (mean ± SD) are provided in Supplementary File S3.

An important aspect of the study was the investigation of physiological changes occurring during growth, with particular emphasis on metabolic processes. Accordingly, we examined phase-specific changes in key blood lipid parameters, including high-density lipoprotein (HDL), low-density lipoprotein (LDL), total cholesterol (CHOL) and triglycerides (TRIGL), in chickens and ducks (Figure 2).

Based on the analysis of continuous changes in lipid fractions (Figure 2a), it can be stated that in chickens (Figure 2(a1)), both CHOL and HDL exhibited a continuous decrease from the starter to the finisher phase, whereas the concentration of LDL showed a slight but consistent increase over the growth phases. In the case of triglycerides (TRIGL), the change was non-linear, with a decrease observed during the grower phase, followed by a moderate increase in the finisher phase, while the values did not reach the levels measured in the starter phase. In ducks (Figure 2(a2)), CHOL decreased from the starter to the grower phase and remained essentially unchanged during the finisher phase. For both HDL and LDL, a decreasing trend was observed up to the grower phase, followed by an increase during the finisher phase. However, in the case of LDL, the concentration measured in the finisher phase exceeded that observed in the starter phase. Triglycerides (TRIGL) showed a distinct pattern, with an increase observed up to the grower phase, followed by a decrease during the finisher phase, while remaining above the values observed in the starter phase. To further assess lipid fraction trends, statistically significant differences in blood lipid parameters between adjacent growth phases were evaluated. In chickens (Figure 2(a1)), no statistically significant differences were observed for any of the examined lipid fractions when comparing adjacent growth phases. In contrast, in ducks (Figure 2(a2)), significant differences were detected between the starter and grower phases for TRIGL (*p* = 0.03) and HDL (*p* = 0.03), whereas no statistically significant differences were observed between the grower and finisher phases for any of the measured parameters.

To complement the trends revealed by the line plots, differences in blood lipid parameters between the starter and finisher phases were also specifically analyzed (Figure 2b). Based on this analysis, HDL (*p* = 0.01) showed a statistically significant difference in chickens (Figure 2(b1)), whereas CHOL (*p* = 0.03) differed significantly in ducks (Figure 2(b2)). Based on the comparison of the poultry species, it can also be stated that most of the examined blood lipid parameters exhibited similar directional changes between the initial and final growth phases. An exception was observed for triglycerides (TRIGL), which showed a decreasing trend in chickens but an increasing trend in ducks during the finisher phase.

**Figure 3:**
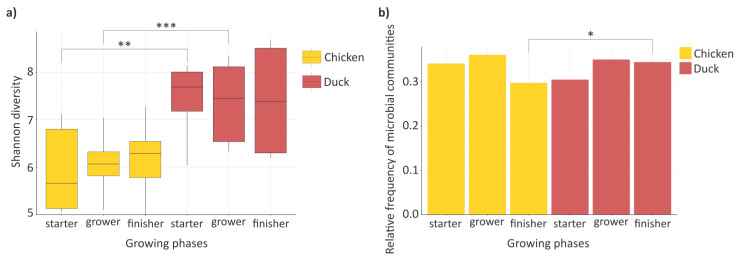
Changes in microbiome diversity and relative abundance across growth phases (**a**) Boxplots illustrating alpha diversity metrics of richness and evenness (Shannon) for chicken and duck samples across the growing phases. (**b**) Barplots showing the relative frequency of bacterial communities in chicken and duck across development. Values represent relative frequencies calculated from mean read counts per group. Asterisks indicate significant differences (* *p* < 0.05, ** *p* < 0.01, *** *p* < 0.001). Color coding is assigned according to avian species, with yellow representing chicken and red representing duck.

In our study, the investigation also included the examination of the Shannon diversity of the poultry flock’s gastrointestinal tract (GIT) (Figure 3a). Changes in the value of this diversity index were examined in relation to the growth of chickens and ducks at different developmental stages. Based on our results, growth did not have a pronounced effect on the diversity indices in the different poultry species (chicken Shannon diversity (mean ± SD), starter 5.93 ± 0.82, grower 6.10 ± 0.51, finisher 6.17 ± 0.63), and although a slight increasing trend can be observed in ducks (duck Shannon diversity (mean ± SD), starter 7.37 ± 0.91, grower 7.39 ± 0.89, finisher 7.41 ± 1.2), this change cannot be considered substantial. However, when the species richness of ducks and chickens was compared within the same growth phases, it was observed that in both the starter (*p* = 0.001) and grower (*p* = 0.001) phases, the diversity values of ducks were significantly higher than those of chickens.

Following the evaluation of the diversity indices, the investigation of the structure of the poultry gastrointestinal microbiome and its changes across developmental stages was continued through the analysis of bacterial relative abundances (Figure 3b). It provides important information on the extent to which the relative proportions of bacterial abundance change across the different growth phases, as well as how these shifts in relative abundance appear during the development of the poultry species. Similar to the diversity indices, when the overall relative frequency was examined across the different phases, no substantial differences were observed within the poultry species, neither in chickens (chicken relative frequency, starter 0.34, grower 0.36, finisher 0.30) nor in ducks (duck relative frequency, starter 0.31, grower 0.35, finisher 0.34). However, when comparing the identical growth phases, it can be stated that in both poultry species the highest value of bacterial relative frequency was measured in the grower phase. In the finisher phase following the grower phase, a greater decrease in relative frequencies was observed in chickens than in ducks, although these changes were not significant in either species. However, a significant difference was already detected between the two species in the values measured in the finisher phase (*p* = 0.028).

**Figure 4:**
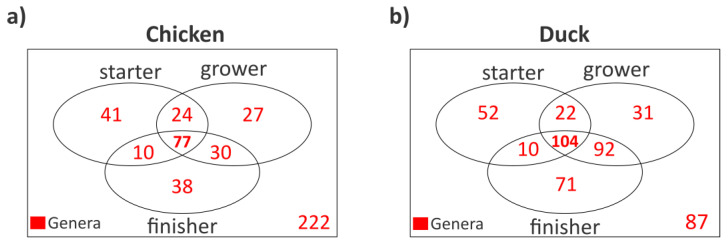
Overlap of genus composition. Venn diagrams illustrate the distribution of genera across growing phases in both chicken (**a**) and duck (**b**). Genera are indicated in red, and core (present in all phases) taxa are highlighted in bold red.

To gain a more precise understanding of the overlap and differences among microbial communities, we also examined how taxa identified at the genus level are distributed across the different growth phases, how many of these are shared among phases or are specific to a given phase (Figure 4). It is noteworthy that in both poultry species the number of genera shared across all three growth phases was the highest (chicken 77, duck 104), indicating that a substantial proportion of microorganisms remains stably present throughout the entire rearing period (Figure 4a,b). This may be particularly significant, as these taxa could play an important role in the early establishment and long-term maintenance of the gut microbiome.

**Figure 5:**
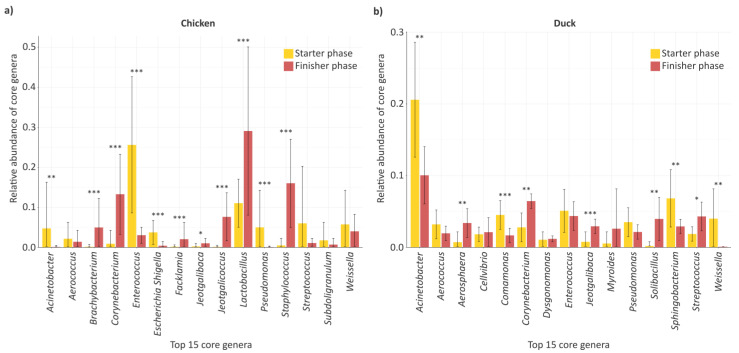
Core microbiome at growth phase endpoints. Barplots illustrate the relative abundance of the top 15 core (100%) genera in chicken (**a**) and duck (**b**) across the starter and finisher growing phases. Asterisks indicate significant differences (* *p* < 0.05, ** *p* < 0.01, *** *p* < 0.001). Color coding distinguishes growing phases, with yellow representing the starter phase and red representing the finisher phase. The relative abundance core values (mean ± SD) are provided in Supplementary File S4.

Following the exploration of genus-level overlaps, a detailed analysis of the core (100%) microbiome components was also conducted, during which the 15 most frequent genera present across all growth phases were identified, and their relative abundances between the starter and finisher phases were examined (Figure 5). In chickens (Figure 5a), among the 15 most frequent genera present across all growth phases, the taxa representing the highest relative abundances were *Lactobacillus*, *Enterococcus*, *Corynebacterium*, *Staphylococcus*, *Jeotgalicoccus*, *Weissella*, *Brachybacterium*, *Aerococcus*, *Pseudomonas*, *Streptococcus*, *Acinetobacter*, *Escherichia*, *Facklamia*, *Jeotgalibaca*, and *Subdoligranulum*. When comparing the growth phases in chickens, it was observed that the relative abundances of the genera *Acinetobacter* (*p* = 0.004), *Enterococcus* (*p* < 0.001), *Escherichia* (*p* < 0.001), and *Pseudomonas* (*p* = 0.001) decreased significantly compared to the starter phase. In contrast, the relative abundances of *Brachybacterium* (*p* < 0.001), *Corynebacterium* (*p* < 0.001), *Facklamia* (*p* = 0.001), *Jeotgalicoccus* (*p* < 0.001), *Jeotgalibaca* (*p* = 0.046), *Lactobacillus* (*p* = 0.001), and *Staphylococcus* (*p* < 0.001) were significantly higher in the finisher phase compared to the starter phase. In ducks (Figure 5b), among the 15 most frequent genera present across all growth phases, the taxa representing the highest relative abundances were *Acinetobacter*, *Sphingobacterium*, *Corynebacterium*, *Enterococcus*, *Pseudomonas*, *Comamonas*, *Aerococcus*, *Streptococcus*, *Cellvibrio*, *Myroides*, *Solibacillus*, *Aerosphaera*, *Jeotgalibaca*, *Dysgonomonas*, and *Weissella*. When comparing the growth phases in ducks, it was observed that the relative abundances of the genera *Acinetobacter* (*p* = 0.003), *Comamonas* (*p* = 0.001), *Sphingobacterium* (*p* = 0.003), and *Weissella* (*p* = 0.009) decreased significantly from the starter to the finisher phase. In contrast, the relative abundances of Aerosphaera (*p* = 0.004), *Corynebacterium* (*p* = 0.002), *Jeotgalibaca* (*p* < 0.001), *Solibacillus* (*p* = 0.001), and *Streptococcus* (*p* = 0.01) were significantly higher in the finisher phase compared to the initial phase.

Following the evaluation of within-species patterns, the extent to which these trends showed similarities or differences between the two poultry species was also examined (Figure 5a,b). In both poultry species, the shared core (100%) genera present across all growth phases were *Acinetobacter*, *Corynebacterium*, *Enterococcus*, *Pseudomonas*, *Aerococcus*, *Streptococcus*, *Jeotgalibaca*, and *Weissella.* For *Acinetobacter*, a decreasing trend was observed in both poultry species, and this change was significant in both cases (*p* < 0.01). The relative abundances of *Enterococcus* and *Pseudomonas* also decreased in both species; however, this reduction was significant only in chickens (*p* < 0.001). In the case of *Weissella*, a decreasing trend was observed in both poultry species; however, a statistically significant change was detected only in ducks (*p* < 0.01). *Aerococcus* exhibited a decreasing tendency in both poultry species, but no significant change was detected in either case. When examining genera showing similar increasing trends, the relative abundances of both *Corynebacterium* (chicken *p* < 0.001, duck *p* < 0.01) and *Jeotgalibaca* (chicken *p* < 0.05, duck *p* < 0.001) were found to increase significantly. For *Streptococcus*, distinct patterns were observed between the two species, with an increase in relative abundance detected in ducks, and this change was found to be significant (*p* < 0.05).

Overall, the trends observed among the shared genera suggest that although the gut microbiomes of the two poultry species exhibit similar patterns at several points, species-specific differences can also be identified for certain taxa during the rearing period (Figure 5a,b). Among these species-specific taxa, those whose relative abundances showed marked changes between the starter and finisher phases in chickens were *Brachybacterium* (*p* < 0.001), Escherichia (*p* < 0.001), *Facklamia* (*p* < 0.001), *Jeotgalicoccus* (*p* < 0.001), *Lactobacillus* (*p* < 0.001), and *Staphylococcus* (*p* < 0.001), whereas in ducks significant changes were observed for *Aerosphaera* (*p* < 0.01), *Comamonas* (*p* < 0.001), *Sphingobacterium (p* < 0.01), and *Solibacillus* (*p* < 0.01).

**Figure 6:**
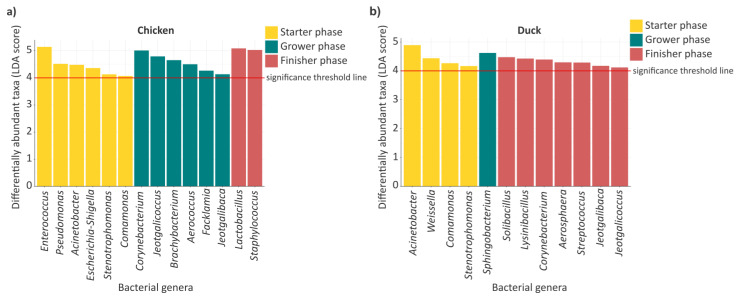
Identified microbial genus-level biomarkers across growing phases in avian species. Linear discriminant analysis (LDA) effect size (LEfSe) identifies bacterial clades involved in significant taxonomic shifts across growing phases, presented separately for chicken (**a**) and duck (**b**). Significance threshold line (LDA > 4) indicates genera that differ significantly across rearing phases. Color coding distinguishes growing phases, with yellow representing the starter phase, green representing the grower phase and red representing the finisher phase.

The analysis of diversity indices, relative abundances, as well as genus-level overlaps and their proportional changes indicated that, although the structure of the gut microbiome exhibited stability in several cases throughout the growth phases, pronounced species-specific differences were observable for certain taxa. Based on these findings, it became justified to examine in greater detail which microbial taxa contribute most prominently to the differences observed among the distinct growth phases and between the two poultry species. To address this, LEfSe (Linear Discriminant Analysis Effect Size) analysis was applied, enabling the identification of biologically distinct taxa with high discriminative power (LDA > 4) across the different growth phases (Figure 6).

In chickens (Figure 6a), the microbial community members characteristically associated with the starter phase included the genera *Enterococcus* (LDA = 5.14), *Pseudomonas* (LDA = 4.52), *Acinetobacter* (LDA = 4.48), *Escherichia* (LDA = 4.36), *Stenotrophomonas* (LDA = 4.13), and *Comamonas* (LDA = 4.07). In contrast, during the grower phase, the genera *Corynebacterium* (LDA = 5.01), *Jeotgalicoccus* (LDA = 4.79), *Brachybacterium* (LDA = 4.65), *Aerococcus* (LDA = 4.50), *Facklamia* (LDA = 4.27), and *Jeotgalibaca* (LDA = 4.13) were distinctly discriminated. Furthermore, it was observed that, despite the exceptionally high LDA values, the finisher phase exhibited the lowest number of prominently represented community members, including the genera *Lactobacillus* (LDA = 5.09) and Staphylococcus (LDA = 5.03). In ducks (Figure 6b), similarly well defined, phase-specific microbial patterns were identifiable. During the starter phase, the genera *Acinetobacter* (LDA = 4.89), *Weissella* (LDA = 4.44), *Comamonas* (LDA = 4.27), and *Stenotrophomonas* (LDA = 4.17) exhibited pronounced differentiation. In contrast, during the grower phase, only a single but distinctly separated taxon was identifiable, namely the genus *Sphingobacterium* (LDA = 4.62). The microbial community composition characteristic of the finisher phase displayed a more diverse profile, in which the genera *Solibacillus* (LDA = 4.47), *Lysinibacillus* (LDA = 4.43), *Corynebacterium* (LDA = 4.39), *Aerosphaera* (LDA = 4.30), *Streptococcus* (LDA = 4.29), *Jeotgalibaca* (LDA = 4.17), and *Jeotgalicoccus* (LDA = 4.12) were significantly differentiated.

Based on the comparison of the two poultry species (Figure 6a,b), it can also be stated that certain microbial genera exhibited significant differentiation simultaneously within the same growth phase in both chickens and ducks. In this context, during the starter phase, the genera *Acinetobacter*, *Comamonas*, and *Stenotrophomonas* were identified, whereas no genera characteristic of both poultry species were identifiable during the grower and finisher phases. In line with this, several microbial genera were also identifiable that occurred in both poultry species but were associated with different growth phases. In all three cases, the genera *Corynebacterium*, *Jeotgalibaca*, and *Jeotgalicoccus* were observed during the finisher phase in ducks, whereas the same taxa were characteristic of the grower phase in chickens.

**Figure 7:**
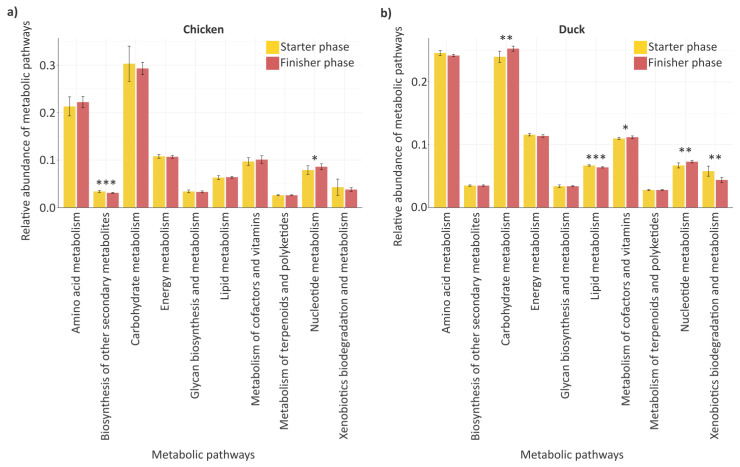
Microbial metabolic pathway profiles. Barplots show the relative abundance of 10 selected microbial metabolic pathways predicted using KEGG functional profiling. Pathway profiles are compared between the starter and finisher phases in chickens (**a**) and ducks (**b**) to evaluate shifts in microbial metabolic potential. The selected pathways represent the most abundant metabolic functions across samples, including amino acid metabolism (AAM), carbohydrate metabolism (CM), lipid metabolism (LM), energy metabolism (EM), nucleotide metabolism (NM), glycan biosynthesis and metabolism (GBM), metabolism of cofactors and vitamins (MCV), biosynthesis of other secondary metabolites (BOSM), metabolism of terpenoids and polyketides (MTP), and xenobiotics biodegradation and metabolism (XBM). Asterisks indicate significant differences (* *p* < 0.05, ** *p* < 0.01, *** *p* < 0.001). Color coding distinguishes growing phases, with yellow representing the starter phase and red representing the finisher phase. The relative abundance values of the metabolic pathways (mean ± SD) are provided in Supplementary File S5.

In relation to the changes observed in the gastrointestinal taxonomic composition and blood lipid parameters of poultry, the predicted functional potential of the gut microbiome was also examined at the level of metabolic pathways. Accordingly, the relative activity of the 10 most abundant microbial metabolic pathways was compared between the starter and finisher phases in order to identify functional shifts associated with growing stages (Figure 7).

In chickens (Figure 7a), two functional pathways showed significant differences during development. The relative abundance of BOSM significantly decreased by the end of the rearing period (*p* = 0.001), whereas NM showed a significant increase (*p* = 0.03). In ducks (Figure 7b), several functional pathways showed significant differences during development. Significant increases were observed in the relative abundance of CM (*p* = 0.003), MCV (*p* = 0.02), and NM (*p* = 0.005), whereas the relative abundance of LM (*p* < 0.001) and XBM (*p* = 0.003) significantly decreased.

During the comparison of the two poultry species (Figure 7a,b), NM was the only functional pathway showing a similar pattern of change, with a significant increase observed in both chickens (*p* < 0.05) and ducks (*p* < 0.01). Similarly, MCV showed an increasing trend in both species. However, the change was not significant in chickens (*p* = 0.3), whereas a significant increase was observed in ducks (*p* < 0.05). A comparable pattern was observed for XBM, as its relative abundance showed a decreasing trend in both chickens (*p* = 0.6) and ducks, with the decrease being significant only in ducks (*p* < 0.01).

In contrast, three functional pathways showed species-specific patterns of change associated with the growing phases, as the direction of the shifts differed between chickens and ducks, and in some cases these differences were significant (Figure 7a,b). The relative abundance of CM showed a decreasing trend in chickens (*p* = 0.2), whereas a significant increase was observed in ducks (*p* < 0.01). An opposite pattern was observed for LM, which showed a significant decrease in ducks (*p* < 0.001), while a slight increase was detected in chickens (*p* = 0.8). The relative abundance of BOSM showed no meaningful change in ducks (*p* = 1), whereas a significant decrease was observed in chickens (*p* < 0.001).

**Figure 8:**
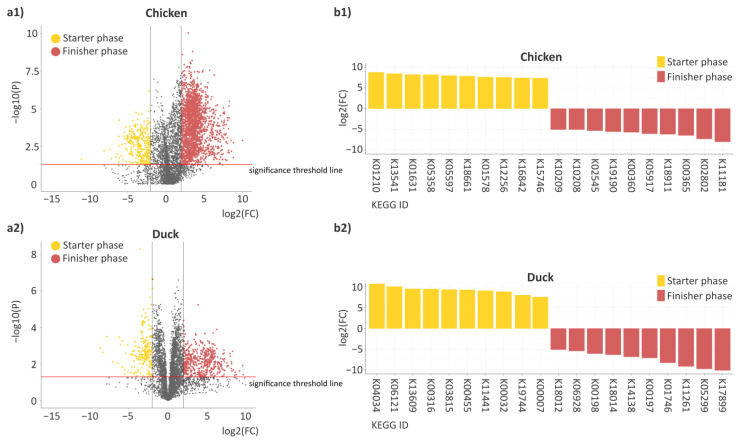
Gene-level functional differences between early and final phases. (**a**) Volcano plots illustrate gene-level functional differences based on predicted KEGG level 3 functional annotations between the starter and finisher phases in chickens (**a1**) and ducks (**a2**). Each point represents a predicted microbial gene plotted by the logarithm of fold change (log2(FC)) and statistical significance. Genes with higher predicted abundance in the starter phase are shown in yellow, whereas those enriched in the finisher phase are shown in red. Gray dots represent genes that did not differ significantly between phases. The x-axis represents the logarithm of fold change (log2(FC)), while the y-axis represents statistical significance expressed as −log10(p). Genes are considered significantly different when log2(FC) > 2 or log2(FC) < −2 and −log10(p) > 1.3. (**b**) The barchart panels summarize the logarithm of fold change values of significantly altered microbial genes with the highest absolute log2(FC) differences between the starter and finisher phases in chickens (**b1**) and ducks (**b2**), highlighting the most pronounced gene-level functional shifts. Positive log2(FC) values indicate higher predicted gene abundance in the starter phase (yellow), whereas negative log2(FC) values indicate higher predicted gene abundance in the finisher phase (red). KEGG ID abbreviations shown on the x-axis are summarized in separate tables for chickens and ducks in the Supplementary File S6.

To complement the taxonomic and metabolic pathway analyses, predicted gene-level functional profiling was performed to assess differences in the abundance of functional genes between the endpoints of the growing phases in poultry (Figure 8). The analysis was based on computational predictions inferred from the identified microbial taxa rather than direct sequencing of functional genes, allowing the estimation of functional genes associated with the different growing phases.

Based on the analysis of the volcano plots, shifts in predicted gene-level functional differences between the starter and finisher phases were observed in both poultry species (Figure 8a). Most significantly different functional genes were associated with the finisher phase. However, in chickens (Figure 8(a1)), a larger proportion of the significantly different genes showed higher predicted abundance in the finisher phase, whereas this shift was less pronounced in ducks (Figure 8(a2)). The functional genes identified in poultry could be grouped into several distinct functional categories (Figure 8b).

In chickens (Figure 8(b1)), several genes related to basic and central metabolic processes showed higher predicted abundance in the starter phase (K01631 log2(FC) = 8.26, K05358 log2(FC) = 8.23, K18661 log2(FC) = 7.87, and K01578 log2(FC) = 7.65). Genes related to nitrogen and amino acid metabolism also showed clear separation between growing phases. Genes enriched in the starter phase included K05597 (log2(FC) = 8.02), K12256 (log2(FC) = 7.57), and K16842 (log2(FC) = 7.46), whereas genes associated with the finisher phase included K18911 (log2(FC) = −6.21) and K00360 (log2(FC) = −5.73). Genes related to cell wall, membrane structures, and the metabolism of specialized molecules also showed phase-specific separation. In the starter phase, K01210 (log2(FC) = 8.79) and K15746 (log2(FC) = 7.39) showed higher predicted abundance, whereas a greater number of differences were associated with the finisher phase. These included genes related to cell wall and transport processes (K02545 log2(FC) = −5.34, K02802 log2(FC) = −7.33), as well as genes involved in the metabolism of specialized molecules (K10208 log2(FC) = −5.08, K10209 log2(FC) = −5.08, K05917 log2(FC) = −6.09, and K19190 log2(FC) = −5.59).

In ducks (Figure 8(b2)), genes related to basic and central metabolic processes showed pronounced phase-specific separation. In the starter phase, several genes related to carbohydrate and energy metabolism showed higher predicted abundance, including K06121 (log2(FC) = 8.62), K11441 (log2(FC) = 7.75), K00032 (log2(FC) = 7.56), K00007 (log2(FC) = 6.46), and K00455 (log2(FC) = 7.94). In contrast, differences associated with the finisher phase primarily involved genes related to central carbon metabolism and anaerobic energy production (K14138 log2(FC) = −5.85, K00197 log2(FC) = −6.07, K00198 log2(FC) = −5.18, K05299 log2(FC) = −8.33, and K11261 log2(FC) = −7.82). Genes related to nitrogen and amino acid metabolism also showed clear separation between growing phases. In the starter phase, genes associated with amino acid and polyamine metabolism showed higher predicted abundance, including K13609 (log2(FC) = 8.15), K00316 (log2(FC) = 8.12), and K19744 (log2(FC) = 6.85). In contrast, differences associated with the finisher phase primarily involved genes related to nitrogen transformation and amino acid degradation (K18012 log2(FC) = −4.36, K18014 log2(FC) = −5.41, K01746 log2(FC) = −7.07, and K17899 log2(FC) = −8.64). For genes related to cell wall, membrane structures, and the metabolism of specialized molecules, higher predicted abundance in the starter phase was observed for K04034 (log2(FC) = 9.18) and K03815 (log2(FC) = 8.00), whereas differences associated with the finisher phase only included K06928 (log2(FC) = −4.64).

**Figure 9:**
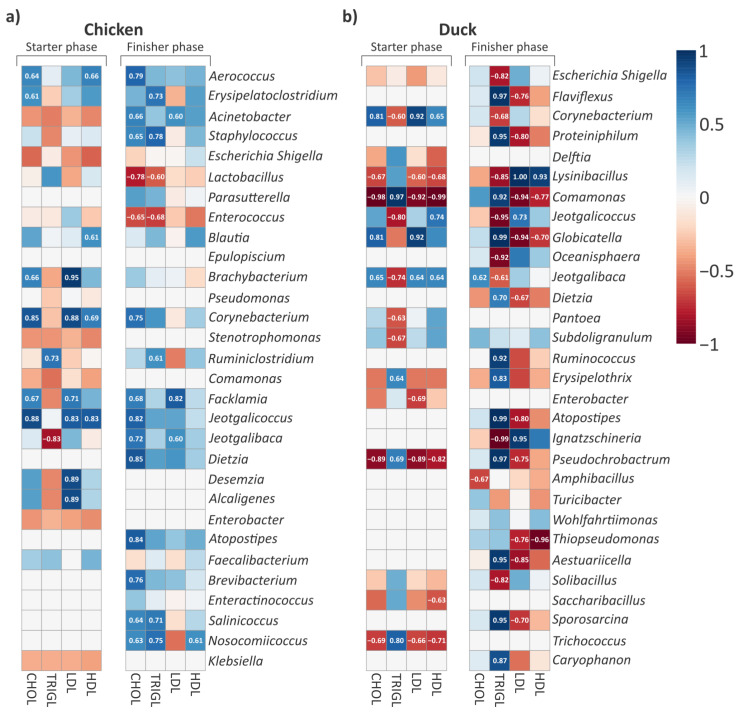
Relationships between host lipid metabolism and dominant gut microbiota. Heatmap of Spearman’s rank correlation coefficients between host lipid parameters (CHOL, TRIGL, LDL, HDL) and the 30 most abundant gut microbial genera selected by LEfSe. The figure compares starter and finisher phases in chickens (**a**) and ducks (**b**). Blue indicates positive correlations, while red indicates negative correlations, with color intensity reflecting the strength of the correlation. Only correlations with r ≥ 0.6 or r ≤ −0.6 are shown.

Based on the differences observed in taxonomic composition, blood lipid parameters, and predicted microbial functions, we further examined whether phase-specific associations could be identified between dominant gut microbial taxa and lipid metabolism parameters in poultry. Accordingly, correlation patterns between dominant gut microbial genera and lipid parameters were analyzed at the endpoints of the growing phases in chickens and ducks (Figure 9).

In chickens, predominantly positive correlations were observed between dominant gut microbial genera and lipid parameters in both the starter and finisher phases (Figure 9a). These correlations were mainly associated with CHOL, LDL, and HDL in the starter phase, whereas associations with triglyceride values were less pronounced. *Aerococcus*, *Brachybacterium*, and *Facklamia* showed positive correlations with CHOL and LDL, whereas *Jeotgalicoccus* and *Corynebacterium* were positively correlated with CHOL, LDL, and HDL simultaneously. In the finisher phase, these positive correlations were primarily associated with CHOL and TRIGL. *Salinicoccus* and *Staphylococcus* showed simultaneous positive correlations with both lipid parameters (CHOL and TRIGL).

In ducks, the correlation patterns between dominant gut microbial genera and lipid parameters were more complex and heterogeneous (Figure 9b). Strong correlations of both positive and negative directions were observed among the lipid parameters. In the starter phase, some genera exhibited distinctly contrasting correlation patterns with lipid parameters. *Corynebacterium* and *Jeotgalibaca* showed strong positive correlations with HDL, LDL, and CHOL, while displaying strong negative correlations with triglyceride values. In contrast, *Trichococcus*, *Pseudochrobactrum*, and *Comamonas* exhibited the opposite pattern, showing strong negative correlations with HDL, LDL, and CHOL and strong positive correlations with TRIGL. In the finisher phase, characteristic opposing correlation patterns were also observed. *Comamonas* and *Globicatella* showed strong negative correlations with LDL and HDL, while exhibiting strong positive correlations with triglyceride values. In contrast, *Lysinibacillus* displayed opposite relationships with the same lipid parameters (LDL, HDL and TRIGL). In addition, several genera were identified, including *Flaviflexus*, *Proteiniphilum*, *Dietzia*, *Atopostipes*, *Pseudochrobactrum*, *Aestuariicella*, and *Sporosarcina*, that showed positive correlations with TRIGL and negative correlations with LDL. In contrast, *Ignatzschineria* and *Jeotgalicoccus* showed negative correlations with triglycerides and positive correlations with LDL.

Eight genera were shared between the dominant gut microbial communities of the two poultry species, including *Escherichia*, *Corynebacterium*, *Comamonas*, *Jeotgalicoccus*, *Jeotgalibaca*, *Dietzia*, *Enterobacter*, and *Atopostipes* (Figure 9a,b). For *Comamonas*, marked differences were observed between the two poultry species in correlations with lipid parameters. For *Corynebacterium*, the correlation patterns in the starter phase showed the same direction in both poultry species (HDL, LDL, and CHOL). Similarly, in the starter phase, *Jeotgalicoccus* showed strong positive correlation with the same lipid parameter in both chickens and ducks (HDL). For *Escherichia* and *Enterobacter*, correlations with lipid parameters were only sporadically observed in both poultry species and did not show a consistent pattern across growing phases.

## 4. Discussion

Intensive poultry production systems provide a controlled framework to examine how host species and growth stage shape the gastrointestinal microbiome and host metabolic status. In this context, understanding whether microbial community structure, predicted functional potential, and lipid metabolism follow species-specific or growth-dependent trajectories is of central importance. Therefore, we investigated broiler chickens and ducks reared under intensive conditions to evaluate how microbiome composition, predicted functional characteristics, and associated lipid parameters vary across the starter, grower, and finisher phases.

Host lipid metabolism exhibited developmental differences across growth phases in both poultry species. In chickens, serum lipid parameters followed relatively stable trajectories. Although CHOL and HDL showed a gradual decline across growth phases and LDL increased slightly, no significant differences were detected between adjacent phases. This pattern is consistent with previous reports indicating that serum triglycerides and cholesterol fractions in broilers often decrease moderately with age or remain relatively stable during the fattening period [23], while HDL levels tend to decline in later growth stages and LDL frequently remains unchanged [24]. Between the starter and finisher phases, HDL decreased significantly (*p* = 0.01). HDL plays a central role in reverse cholesterol transport and redistribution of lipids from peripheral tissues to the liver [25]; therefore, its reduction toward the finisher phase may reflect maturation-associated shifts in lipid utilization and lipoprotein dynamics during late growth. In ducks, lipid parameters showed more pronounced early-phase dynamics, with HDL decreasing (*p* = 0.03) and triglycerides increasing significantly (*p* = 0.03) between the starter and grower phases, reflecting active metabolic adjustment during early growth. These findings suggest modulation of hepatic lipid synthesis and lipoprotein transport during the transition from early growth to more intensive tissue deposition. Similar early-life lipid remodeling has been reported in ducks, where cholesterol fractions change dynamically during growth [26]. Between the starter and finisher phases, total cholesterol differed significantly (*p* = 0.03), indicating growth-associated changes in cholesterol homeostasis. As circulating cholesterol levels are influenced by genetic and broader external factors, this decline likely reflects physiological adjustment during development [25].

Microbial diversity contributes to ecosystem stability within the host, supports nutrient utilization, immune modulation, and overall host health [27]. In the present study, no significant differences were observed in diversity indices within species, indicating a stable and well-balanced microbial community. Shannon diversity and relative abundance patterns suggest that gastrointestinal microbiome structure in poultry is shaped primarily by species-specific host factors rather than growth stage. The relative stability of diversity across developmental phases in both chickens and ducks further supports the limited influence of age under standardized environmental and dietary conditions, consistent with previous reports [28], and with findings demonstrating minimal intra-species variation but persistent inter-species differences [29]. Ducks consistently exhibited higher microbial diversity, particularly during the starter (*p* = 0.001) and grower (*p* = 0.001) phases, likely reflecting inherent host-related traits that support a more heterogeneous gut ecosystem [29]. In contrast, the reduction in bacterial abundance observed in chickens during the finisher phase (*p* = 0.028) suggests stronger host-mediated constraints on microbiome structure during late growth.

The persistence of a stable core microbiome across all growth phases in both species underscores the importance of early-life colonization in establishing long-term community members [30]. Similar to other studies demonstrating age-related microbiota succession, the reduction in facultative or environmentally associated taxa such as *Acinetobacter* in both chickens (*p* = 0.004) and ducks (*p* = 0.003), as well as *Enterococcus* in chickens (*p* < 0.001), from starter to finisher phases in our data supports a dynamic maturation process, where early colonizers are gradually replaced or outcompeted by more specialized, host-adapted microbes as the gut environment becomes more anaerobic and functionally differentiated with age [31]. Furthermore, the increase in the relative abundance of *Corynebacterium* in chickens (*p* < 0.001) and ducks (*p* = 0.002), as well as *Lactobacillus* in chickens (*p* = 0.001), aligns with the well-documented roles of these taxa in carbohydrate fermentation, short-chain fatty acid production, and epithelial interaction functions that become increasingly important during later stages of intestinal development [32]. In addition, the significant decline of *Escherichia* (*p* < 0.001) and *Pseudomonas* (*p* = 0.001) in chickens further reflects the transition from early facultative colonizers toward a more stable, anaerobe-dominated intestinal ecosystem, a pattern widely described in age-related microbiota succession in poultry [4]. The observation of similar abundance trends across multiple genera (*Acinetobacter*, *Enterococcus*, *Pseudomonas*, *Aerococcus*, *Corynebacterium*, *Jeotgalibaca*, *Weissella*) in both chickens and ducks suggests shared developmental mechanisms governing microbiome maturation, likely reflecting common physiological processes such as increasing gut anaerobiosis, immune maturation, and stabilization of intestinal niches [31,33,34].

LEfSe analysis further highlighted distinct successional trajectories. The enrichment of *Acinetobacter*, *Comamonas*, and *Stenotrophomonas* during the starter phase in both species is consistent with early dominance of facultative and environmentally associated taxa that decline as the gut environment stabilizes [35]. In chickens, the progressive enrichment of *Corynebacterium*, *Jeotgalicoccus*, and related genera during the grower phase suggests a transitional restructuring stage, while the dominance of *Lactobacillus* in the finisher phase aligns with its well-established role as a hallmark of mature poultry microbiomes [36,37]. In ducks, the broader and delayed emergence of finisher-associated biomarkers reflects species-specific microbiome succession dynamics, consistent with reports of higher environmental responsiveness in duck gut communities [38].

To better understand the functional differences observed between the starter and finisher phases, we examined whether genera identified by LEfSe were consistent with the predicted metabolic pathways. In both species, starter-phase-associated communities were characterized by metabolically flexible and environmentally associated taxa. In chickens, genera enriched during the starter phase included *Escherichia*, *Enterococcus*, *Pseudomonas*, and *Acinetobacter*, while in ducks, *Acinetobacter*, *Comamonas*, and *Stenotrophomonas* were predominant. These taxa are commonly linked to competitive and secondary metabolic activities, corresponding with the higher relative abundance of BOSM and XBM in the starter phase in both species. The phase difference was significant for BOSM in chickens (*p* = 0.001) and for XBM in ducks (*p* = 0.003). In contrast, the finisher phase was characterized by enrichment of genera associated with host-adapted and growth-related metabolism. In chickens, *Lactobacillus* was dominant, whereas in ducks, *Streptococcus* and *Corynebacterium* were characteristic. The enrichment of these genera was consistent with the observed increase in NM in both species, which was significant in chickens (*p* = 0.03) and in ducks (*p* = 0.005), suggesting enhanced microbial proliferation and metabolic activity during the finisher phase. Regarding the predicted metabolic functions of the gut microbiome in chickens, only a limited number of pathways changed significantly across growth phases, suggesting relatively stable functional organization. The increase in NM (*p* = 0.03) alongside reduced BOSM (*p* = 0.001) during the finisher phase is consistent with a transition toward a stable, low-competition microbiome optimized for efficient nutrient utilization [39]. By contrast, ducks exhibited more extensive functional shifts, suggesting greater flexibility of the gut microbiome during growth. The increased contribution of CM (*p* = 0.003) implies improved microbial capacity for energy harvest peaking in the mid-to-late growth stages, whereas the rise in NM (*p* = 0.005) at later stages alongside overall restructuring reflects adaptations to protein synthesis demands, as also reported by Ma [40]. To further support these functional interpretations, the dominant genera were evaluated in the context of their known metabolic capabilities. The enrichment of *Pseudomonas* and *Enterococcus* aligns with their known roles in secondary metabolite production and microbial competition. As demonstrated by Silby [41], *Pseudomonas* species exhibit remarkable metabolic and physiological versatility, producing a wide range of bioactive secondary metabolites that contribute to niche establishment. Similarly, Umu [42] reported the production of Class II bacteriocins by *Enterococcus*, which can effectively modulate gut microbial communities by suppressing competing taxa. Furthermore, the presence of *Acinetobacter* is consistent with functional pathways related to lipid metabolism and xenobiotic degradation. Dahal [43] characterized *Acinetobacter* as a metabolically versatile genus with broad capabilities for the degradation of complex compounds, including hydrocarbons, supported by its lipolytic activity. In addition, the dominance of *Lactobacillus* corresponds with increased nucleotide metabolism, a process essential for microbial growth and proliferation. As reported by Kilstrup [44] nucleotide metabolism LAB, nucleotides play a central role in nucleic acid synthesis and cellular energy transfer, thereby supporting the metabolic demands of rapid microbial expansion.

Functional differences at the gene level between the starter and finisher phases indicate that microbiome development is strongly influenced by species-specific host pressures. In chickens, intensive selection for rapid growth and feed efficiency appears to restrict microbial functional potential, resulting in a finisher-phase microbiome enriched in genes related to cell wall synthesis, transport mechanisms, and specialized metabolic pathways (e.g., KEGG K02545, K02802, K10208), as also suggested by Cheng [45]. These functions are associated with membrane integrity, substrate transport, and metabolite processing, contributing to microbial stabilization and optimized nutrient utilization in a tightly regulated intestinal environment [45,46]. In contrast, the starter phase showed a higher abundance of genes involved in core metabolic processes, including central carbon and nitrogen metabolism (e.g., K01631, K05358), indicating broadly active metabolic functions during early colonization that decline as the gut ecosystem matures [39]. In ducks, which have experienced comparatively weaker artificial selection, gene-level shifts indicate a more metabolically adaptable microbiome. During the starter phase, enrichment of genes related to carbohydrate utilization and energy metabolism (e.g., K06121, K00032, K11441) likely supports early microbial establishment [36,47]. In the finisher phase, increased representation of genes linked to central carbon metabolism, anaerobic energy generation, and nitrogen transformation (e.g., K05299, K00197, K17899) reflects functional reorganization in response to changing host metabolic demands and nutrient availability. These patterns suggest that chicken microbiomes are shaped toward functional efficiency and stability, whereas duck microbiomes retain greater metabolic flexibility throughout development.

Finally, the observed correlations between dominant gut bacterial genera and host lipid parameters highlight lipid metabolism as a key driver of species- and growth phase-specific host microbiome interactions. In chickens, positive associations of *Corynebacterium*, *Jeotgalicoccus*, and *Facklamia* with CHOL and LDL suggest a host-regulated microbial ecosystem linked to lipid utilization under high-energy diets, while negative correlations of *Lactobacillus* and *Enterococcus* with cholesterol and triglycerides in the finisher phase indicate modulation of lipid homeostasis. This is supported by Deng, who demonstrated that dietary supplementation with *L. acidophilus*, *L. plantarum*, and *E. faecium* reduced serum cholesterol and triglycerides in laying hens through HMGR pathway downregulation and enhanced bile acid excretion [48]. Ducks exhibited more heterogeneous correlation patterns, with *Comamonas* and *Pseudochrobactrum* showing positive associations with triglycerides and negative associations with cholesterol parameters, while *Corynebacterium* and *Jeotgalibaca* maintained conserved positive lipid correlations. Previous studies describing a dynamic and environmentally responsive caecal microbiome in Pekin ducks are consistent with this interpretation [38]. Overall, the results indicate a more synchronized microbiome–lipid association pattern in chickens, whereas ducks display greater variability in microbiome–lipid relationships across development. The gut microbiota plays a central role in regulating host immune function and inflammatory responses. Microbial communities influence immune homeostasis, intestinal barrier integrity, and systemic inflammation through the production of metabolites and modulation of host signaling pathways [49,50,51]. Previous studies have demonstrated that specific microbial taxa and their metabolic activity can shape inflammatory processes and immune responses, highlighting the close link between microbiome composition and host physiology [49,50,51]. In this context, the observed shifts in microbiome composition and functional potential across growth phases may also have implications for host immune regulation, further emphasizing the integrative role of the gut microbiome in coordinating metabolic and physiological processes [49,51,52].

## 5. Conclusions

Under intensive rearing conditions and standard management, microbial diversity remained broadly stable across growth phases, while being primarily influenced by species-related and environmental factors. In ducks, predicted functional profiles showed greater variability, indicating higher functional flexibility and a more dynamic gut microbiome during growth, which was paralleled by more dynamic changes in blood lipid profiles. This greater variability was also reflected in more heterogeneous and phase-dependent microbiome–lipid associations.

Microbiome composition and blood lipid profiles derived from intensive production systems provide practical insight into host–microbiome interactions under commercial conditions. However, as functional pathways and gene-level profiles were predicted from 16S rRNA data, further targeted validation is required for more robust conclusions. Moreover, this study was conducted at a single production site, which may limit the generalizability of the findings across different geographic regions. Nevertheless, all animals were reared under intensive and highly standardized conditions, including controlled housing and management practices, which likely reduce environmental variability and location-specific effects.

## Data Availability

All sequence data used in the analyses were deposited in the Sequence Read Archive (SRA) (http://www.ncbi.nlm.nih.gov/sra, accessed on 13 April 2026) under PRJNA1436209.

## Supplementary Materials

The following supporting information can be downloaded at: https://www.mdpi.com/article/10.3390/ani16081240/s1. The nutritional composition of the feed is provided in Supplementary File S1 for detailed reference. The raw blood parameter data are provided in Supplementary File S2. The summary blood parameter values (mean ± SD) shown in Figure 2 are provided in separate tables for chickens and ducks in Supplementary File S3. The relative abundance core values and their corresponding standard deviations shown in Figure 5 are provided in separate tables for chickens and ducks in Supplementary File S4. The relative abundance values of the metabolic pathways (Figure 7) and their corresponding standard deviations are provided in Supplementary File S5. The KEGG ID abbreviations shown on the x-axis (Figure 8b) are summarized in separate tables for chickens and ducks in Supplementary File S6.
